# Slot-Waveguide Silicon Nitride Organic Hybrid Distributed Feedback Laser

**DOI:** 10.1038/s41598-019-54655-4

**Published:** 2019-12-05

**Authors:** Florian Vogelbacher, Martin Sagmeister, Jochen Kraft, Xue Zhou, Jinhua Huang, Mingzhu Li, Ke-Jian Jiang, Yanlin Song, Karl Unterrainer, Rainer Hainberger

**Affiliations:** 1 AIT Austrian Institute of Technology GmbH, Center for Health & Bioresources, Giefinggasse 4, 1210 Vienna, Austria; 2 TU Wien, Photonics Institute, Gusshausstraße 27–29, 1040 Vienna, Austria; 3 ams AG, Tobelbader Straße 30, 8141 Premstätten, Austria; 4 Institute of Chemistry, Chinese Academy of Sciences, Beijing, 100190 P.R. China

**Keywords:** Solid-state lasers, Integrated optics

## Abstract

One of the major barriers for a widespread commercial uptake of silicon nitride photonic integrated circuits for cost-sensitive applications is the lack of low-cost monolithically integrated laser light sources directly emitting into single-mode waveguides. In this work, we demonstrate an optically pumped organic solid-state slot-waveguide distributed feedback laser designed for a silicon nitride organic hybrid photonic platform. Pulsed optical excitation of the gain medium is achieved by a 450 nm laser diode. The optical feedback for lasing is based on a second-order laterally coupled Bragg grating with a slot-waveguide core. Optimized material gain properties of the organic dye together with the increased modal gain of the laser mode arising from the improved overlap of the slot-waveguide geometry with the gain material enable single-mode lasing at a wavelength of 600 nm. The straightforward integration and operation with a blue laser diode leads to a cost-effective coherent light source for photonic integrated devices.

## Introduction

Photonic integrated circuits (PICs) are widely employed in the field of data- and telecommunication^1^, and have gained much attention in various other fields of application such as bio-medical and chemical sensing^2,3^, optical gyroscopes^4,5^, optical coherence tomography^6,7^, and astronomy^8^. Most of these PICs rely either on an external coupling of coherent light into the chip, which can be achieved for example by end-facet^9^ or grating coupling^10,11^, or the heterogeneous integration of a semiconductor laser through bonding. Therefore, commonly employed coherent light sources require additional fabrication steps or advanced packaging technologies to be used in an integrated photonic platform. For low-cost applications in single-use products an alternative scheme is mandatory. A potential substitute for semiconductor laser diodes are organic solid-state lasers (OSSLs)^12^. However, despite strong research efforts and the tremendous success in organic light-emitting diodes (OLEDs), electrically driven OSSLs are difficult to realize. Indication of lasing for a current injected OSSL has been recently shown for a surface emitting device^13^. In general, OSSLs are optically pumped to achieve population inversion for lasing^12,14^. These lasers show low threshold^15–17^, high peak power^18^, and can cover the visible to near infrared wavelength region^12,18^. The possibility to locally deposit the gain medium reduces material costs and enables the co-integration with other functional devices on a single PIC^19–21^. While in most cases costly solid-state laser pump sources are employed for optical excitation of OSSLs, inexpensive semiconductor laser diodes (LDs) and light-emitting diodes (LEDs) have also been successfully demonstrated as a viable alternative^17,21–23^. Optical feedback for OSSLs has been achieved by a variety of cavity geometries. Early OSSLs were realized by bulk optics^24^, but soon alternative designs have been developed. For example, vertical emission has been realized with vertical cavity surface-emitting lasers (VCSELs)^25–27^, vertical external cavity surface-emitting organic laser (VECSOL)^28^, and Bragg gratings^17,22,29–31^. However, the in-plane emission of the laser radiation into planar waveguides is a prerequisite for the application as an integrated light source in PIC devices. Additionally, many components of a PIC rely on single-mode waveguides to avoid beating effects between modes of different order. This is especially true when interferometric sensors, such as a Mach-Zehnder interferometer (MZI)^32,33^, are employed. Alongside the exclusive usage of organic materials for an all-organic PIC platform, there is the option for a hybrid integration based on the joint use of organic and inorganic materials. For example, Korn *et al*. demonstrated an integrated silicon-organic hybrid (SOH) laser for a laser emission wavelength at 1310 nm employing the back reflection from a cleaved facet and an on-chip grating coupler, and proposed the combination of a Bragg reflector with a slot-waveguide geometry^18^. Such waveguide Bragg gratings with slot-waveguide geometry have been successfully applied in electro-optic modulators^34^ and sensing devices^35^.

For operation wavelengths in the visible to near-infrared silicon nitride (SiN) is well established as a low-loss photonic integrated platform^3,36^. Low-temperature silicon nitride deposited by plasma enhanced chemical vapor deposition (PECVD) allows the back-end-of-line monolithic co-integration of the optical waveguides with electronics, i.e. together with complementary metal-oxide-semiconductor (CMOS) technology. This combination enables new PIC applications in which sensing, signal processing, and data transmission can be jointly integrated on a single chip. Even though waveguide propagation losses are usually larger when a PIC is fabricated with a PECVD process compared to high temperature alternatives^37,38^, they are commonly below $2\,{{\rm{d}}{\rm{B}}{\rm{c}}{\rm{m}}}^{-1}$^36^. Additionally, the moderate refractive index contrast between SiN and a silicon dioxide (${{\rm{SiO}}}_{2}$) cladding leads to optical waveguide components with feature sizes that can be well resolved by standard photolithography. Hybrid combinations of silicon nitride and dye-doped polymers for an integrated organic solid-state laser have recently been demonstrated for spirals and Bragg gratings^21,39^.

In this work, we demonstrate an integrated organic-solid-state laterally coupled slot-waveguide distributed feedback (DFB) laser on a silicon nitride photonic platform. This SiN-organic hybrid (SiNOH) coherent light source was designed for single-mode emission at a wavelength of 600 nm, which is a highly interesting wavelength for biosensing applications because of the reduced absorption in aqueous solutions^3,40^ and the increased sensitivity^32,33^ for Mach-Zehnder interferometers compared to longer wavelengths. The slot-waveguide structure increases the modal gain compared to a strip-waveguide, accordingly lowering the lasing threshold by increasing the overlap with the gain material. The slot-waveguide laser emits the laser light into a single-mode waveguide, enabling a direct application of the light source in the PIC. The presented laser does not rely on the back-reflection of a cleaved facet and can therefore be readily integrated into a PIC design. Optical excitation was achieved by a pulsed 450 nm blue laser diode. The fabrication of the SiNOH integrated light source is compatible with standard CMOS semiconductor fabrication processes and enables the potential co-integration with electronics to create active sensing devices.

## Results

### Slot-waveguide SiNOH laser design

**Figure 1:**
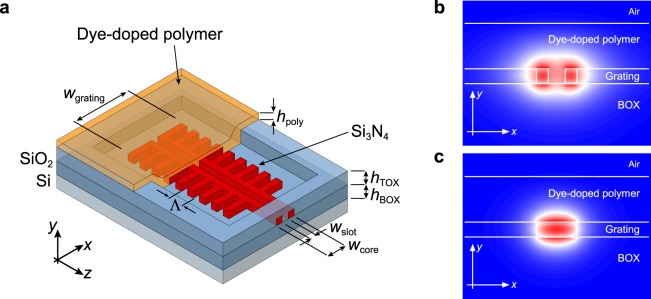
(**a**) Schematic drawing of the laterally coupled slot-waveguide SiNOH DFB laser feedback structure. The dimensions of the slot-waveguide are ${h}_{{\rm{WG}}}=160$ nm, ${w}_{{\rm{core}}}=440$ nm, and ${w}_{{\rm{slot}}}=180$ nm. The grating period is $\Lambda =387$ nm with a duty cycle of $20 \%$. The corrugation of the DFB (${w}_{{\rm{g}}{\rm{r}}{\rm{a}}{\rm{t}}{\rm{i}}{\rm{n}}{\rm{g}}}=4$ µm) is large compared to the lateral dimension of the guided mode. The dye-doped polymer matrix (${h}_{{\rm{poly}}}=500$ nm) provides the modal gain for lasing. Only the DFB structure is exposed to the gain material through a local opening of the top oxide (TOX), which ensures low propagation loss of the laser emission without re-absorption. (**b**) Electric field component ${E}_{y}$ of the fundamental TM-like mode for slot-waveguide and comparable (**c**) strip-waveguide geometry. The modal gain is increased for a slot-waveguide design by the improved interaction of the waveguide mode with the gain material.

Figure 1a depicts the general structure of the laterally coupled slot-waveguide distributed feedback SiNOH laser. The lateral corrugation allows a single photolithography and etching step during fabrication of the planar silicon nitride structures. The SiN waveguide is separated from the silicon substrate by 5 µm buried oxide (BOX). The grating width ${w}_{{\rm{grating}}}$ of the DFB is 4 µm and therefore large compared to the lateral dimension of the guided mode. In contrast to shallow lateral corrugations, this design exhibits a strong coupling between the forward- and backward-propagating cavity modes to enable lasing with the power and pump length limited beam of the laser diode pump source. The gain material has local access to the silicon nitride DFB laser cavity through an opening in the top oxide (TOX) layer.

The introduction of a slot for the laterally coupled DFB resonator increases the overlap between the laser mode and the gain medium, resulting in an increased modal gain. Even though the TE-like mode is usually employed in slot-waveguide applications because of the field enhancement in the slot, lasing in the TM-like mode is favoured over the TE-like mode in the presented geometry because of lower propagation losses and better coupling to the grating. The fraction of mode power in the gain material is given by

1 $$\eta =\frac{{\iint }_{{\rm{gain}}}{P}_{z}\ dA}{{\iint }_{\infty }{P}_{z}\ dA}$$

where ${P}_{z}$ denotes the Poynting vector in the direction of propagation of the waveguide mode. The electromagnetic field distributions of a slot- and strip-waveguide in TM-like polarization are depicted in Fig. 1b,c, respectively. The fraction of mode power in the gain material increases from ${\eta }_{{\rm{strip}}}=28 \%$ for the strip-waveguide to ${\eta }_{{\rm{slot}}}=47 \%$, respectively. A slot-to-strip taper is employed to convert between the slot-waveguide laser resonator mode and the stripe-waveguide mode^41^. For increased sensitivity in biosensing applications the slot-waveguide mode can be directly employed omitting the slot-to-strip taper^32^. The cross section of the SiN strip-waveguide to route the laser emission to the sample edge is $550\,{\rm{n}}{\rm{m}}\times 160\,{\rm{n}}{\rm{m}}$.

**Figure 2:**
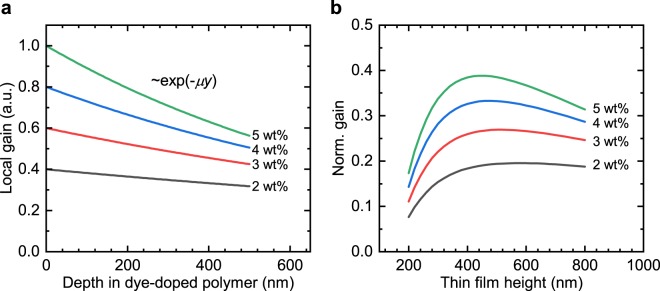
Optimization of optical gain. (**a**) Dependence of the local gain on the vertical position within the thin film for different dye concentrations. The local gain is normalized to the local gain of $$5$$ wt% PMN:PMMA at the dye-doped polymer surface. (**b**) Dependence of the modal gain on the thickness of the dye-doped polymer film. The absorption of the pump light leads to a spatial variation of the local gain in the film. For film thicknesses $$ > 500$$ nm the local gain of the PMN-doped polymer close to the silicon nitride waveguide surface is significantly reduced and thicker layers of gain material are detrimental to the modal gain. The results are based on eigenmode calculations assuming a local gain dependence $$g\propto \exp (-\mu y)$$ inside the gain material layer.

The pump light is absorbed by the gain material, which leads to a gain profile inside the dye-doped polymer layer. If the gain material layer is too thick, only a small fraction of the pump light will reach the gain material nearby the silicon nitride waveguide resonator structure and the local gain in the evanescent field of the guided mode will be small. On the other hand, for a gain layer that is too thin the overlap between the guided mode and the gain material is small because the field is mainly located in the substrate. If a spatial dependence of the pump light intensity is assumed to follow the Beer–Lambert–Bouguer law ${I}_{{\rm{pump}}}\propto \exp (-\mu y)$ and saturation effects of the gain material are neglected for the pump intensities present in these experiments, the local gain follows the same exponential relation (see Fig. 2a). An optimal thickness has been numerically determined with eigenmode calculations. For the deployed dye-doped polymer PMN:PMMA at a doping level of $5.0$ wt%, the value of the decay constant was determined to $\mu =1.15\times {10}^{4}\,{{\rm{c}}{\rm{m}}}^{-1}$. The influence of the polymer film thickness on the modal gain of the TM-like guided mode is shown in Fig. 2b for different dye concentrations $c$, where the relation $\mu \propto c$ was assumed. The largest modal gain is achieved for a film thickness of 400–500 nm.

The grating period $\Lambda$ of a Bragg grating of order $N$, effective refractive index ${n}_{{\rm{eff}}}$, and resonance wavelength ${\lambda }_{0}$ is given by

2 $$\Lambda =\frac{{\lambda }_{0}}{2{n}_{{\rm{neff}}}}N.$$

**Figure 3:**
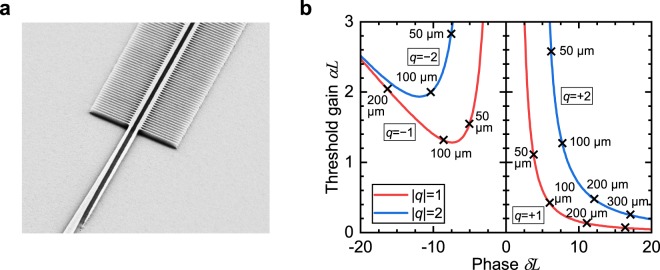
(**a**) Scanning electron micrograph of laterally coupled silicon nitride slot-waveguide DFB structure and the slot-waveguide taper. The grating width is 4 µm and the grating period 387 nm. (**b**) Numerically calculated threshold gain and deviation from of a second-order laterally coupled slot-waveguide DFB with varying length $L$. The lengths of the DFB resonators, ranging from 50 µm–300 µm, are given next to the curve. The threshold gain was determined by coupled wave theory including higher order partial waves to account for radiative modes. The radiative modes lead to a longitudinal mode discrimination. Lasing commences for the mode with the lowest threshold gain.

A second-order Bragg grating with a grating period of 387 nm was implemented to achieve lasing at a wavelength of 600 nm. A scanning electron micrograph of the fabricated structure is shown in Fig. 3a. The use of a second-order grating enabled a fabrication with deep-UV photolithography. In contrast to a first-order grating, where a phase shift of $\lambda /4$ is commonly included into the DFB resonator to provide mode discrimination, the out-of-plane first-order radiation of a second-order Bragg grating leads to an intrinsic mode selection and no phase element is necessary for single-mode operation^42,43^. The in-phase or out-of-phase radiation from the forward and backward propagating traveling waves enhances or suppresses radiative loss, respectively. The suppression of first-order diffraction radiative loss for one mode enables an efficient in-plane emission into the single-mode waveguide, while at the same time the complementary longitudinal mode experiences increased losses leading to the mode discrimination^42^.

The numerically determined dependence of the threshold gain $\alpha$ and the deviation from the Bragg condition $\delta =\beta -{\beta }_{0}$ for the SiNOH slot-waveguide DFB laser in TM-like polarization on the resonator length $L$ is presented in Fig. 3b. For short cavities the threshold gain is high because of the significant under-coupling of the forward and backward propagating cavity modes compared to the length of the cavity. In this case, the major loss components to overcome arise from the cavity end faces. The threshold gain drops for an increasing cavity length, resulting in 2.2 cm${}^{-1}$ at a length of 300 µm for the $q=+\,1$ longitudinal cavity mode. A strong asymmetry in threshold gain between the $q=+\,1$ and $q=-\,1$ longitudinal cavity mode can be observed, which results in a good mode selection from the first-order radiation^42,44^. The second higher cavity mode $q=+\,2$ has a theoretical threshold gain of 8.3 cm${}^{-1}$. The threshold gain calculations do not include additional losses, e.g. arising from incoherent scattering. The propagation loss of the TE-like slot-waveguide mode is approximately $5$ dB/cm higher than for the TM-like mode, resulting in a mode selection favouring the TM-like lasing mode. The material gain needed to reach threshold is given by ${g}_{{\rm{thr}}}/\eta$, where $\eta$ denotes the optical power overlap in Eq. (1) between the guided mode and the gain medium.

### Organic gain material

**Figure 4:**
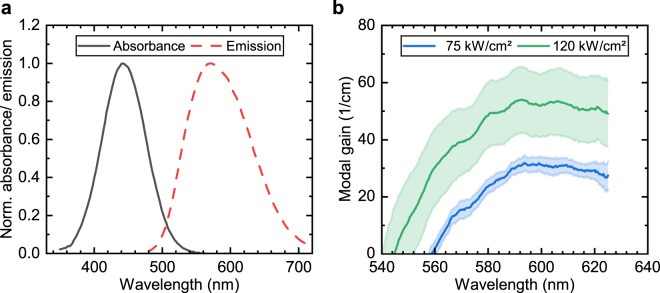
Optical properties of the gain material PMN:PMMA. The doping level of PMN embedded in PMMA was $5.0$ wt%. (**a**) Normalized absorbance and emission spectrum. The excitation wavelength for the measurement of the fluorescence spectrum was set to the wavelength of the optical pump laser diode of 450 nm. (**b**) Spectral modal gain of the slot-waveguide covered with a 500 nm thick gain material layer. The spectral gain was determined with the variable stripe length method. The bands indicate the $95 \%$ confidence intervals.

The organic dye molecule 2-(4-(bis(4-(*tert*-butyl)phenyl)amino)benzylidene)malononitrile (PMN) was directly embedded into the host polymer poly(methyl methacrylate) (PMMA). This dye-doped polymer was used as the gain material to achieve lasing. The doping level of PMN in PMMA was set to $5.0$ wt%, which optimizes the material gain for the specific dye and host polymer^45^. Higher concentrations lead to material gain concentration quenching caused by the interaction between individual dye molecules. Figure 4a depicts the absorbance and emission spectra of the dye-doped polymer. The gain material shows a peak absorbance at 442 nm, which is very close to the emission wavelength of 450 nm of the pump laser diode, enabling an efficient absorption of the pump light. The dye exhibits a large Stokes shift of 128 nm with a peak fluorescence emission wavelength of 570 nm. For gain measurements the variable stripe length (VSL) method was employed. In these measurements, the slot-waveguide was covered with a gain material layer of 500 nm thickness. The deduced spectral modal gain reached up to 32 cm${}^{-1}$ at a pump intensity of 75 kW cm${}^{-2}$, and 54 cm${}^{-1}$ at 12 kW cm${}^{-2}$, respectively. The polarization of the pump light was set perpendicular to the waveguide. Figure 4b depicts the obtained spectral modal gain. In general, the broad spectral gain of organic dyes enables a wide tuning of the OSSL emission wavelength. For example, a tuning of more than 74 nm has been obtained with the employed PMN doped PMMA for a binary Bragg grating structure^21^. The straightforward relationship between the grating period $\Lambda$ and the lasing wavelength ${\lambda }_{0}$ given in Eq. (2) can be used to provide coherent light sources for wavelength multiplexing on lab-on-chip devices.

### SiNOH laser properties

**Figure 5:**
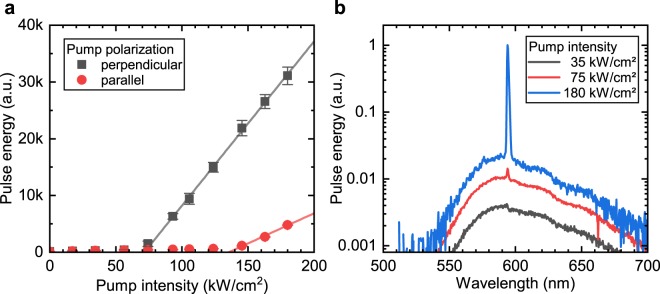
Emission properties of slot-waveguide SiNOH DFB laser. The laser structure was covered with a 500 nm thick gain material (PMN:PMMA, $5.0$ wt%) layer. The emitted laser light coupled to the SiN single-mode waveguide was collected with a multimode fiber at the facet of the PIC. The maximum pump intensity obtained with the blue pump laser diode amounted to 180 kW cm${}^{-2}$ at an optical pulse width of 35 nm. (**a**) Laser threshold measurements for parallel and perpendicular pump polarizations. (**b**) Output emission spectra below, at, and above threshold for perpendicular pump polarization.

In the following we present the emission properties of the SiNOH integrated light source and verify lasing based on the criteria formulated by Samuel *et al*.^46^. Figure 5a shows the determined lasing thresholds for the two major pump polarizations, i.e. parallel and perpendicular relative to the long axis of the laser cavity. The lasing threshold was found to be (72 ± 1) kW cm${}^{-2}$ for the perpendicular pump polarization and (106 ± 7) kW cm${}^{-2}$ for the longitudinal pump polarization, respectively. From coupled wave theory calculations for the laterally coupled DFB cavity presented above, the laser threshold at one edge of the bandgap was estimated to be 2.2 cm${}^{-1}$, which is readily achieved by the employed gain material. However, the actual lasing threshold at 72 kW cm${}^{-2}$ corresponds to a modal gain of approximately 32 cm${}^{-1}$, and therefore is significantly higher than expected from the CWT calculations. This indicates additional loss paths that are not taken into account in the theoretical model. These additional loss paths increase the gain threshold in the experiment above the theoretical value. The lasing threshold of the laterally coupled slot-waveguide SiNOH DFB laser is approximately one magnitude higher than that of a binary grating SiNOH laser^21^. Optimization of the fabrication and design offer improvements to lower the lasing threshold.

Figure 5 summarizes the normalized emission spectra above, below, and at threshold. Broadband fluorescence is observed below the threshold, whereas, above threshold a narrow band emission peak is observed. The side mode suppression is better than 15 dB at a pump intensity of 180 kW cm${}^{-2}$. In contrast to surface emitting gratings, where the background fluorescence intensity decreases inversely proportionally to the square of the distance, the integrated slot-waveguide structure couples fluorescence light into the waveguide, which is efficiently routed to the end facet of the PIC and leads to a higher fluorescence background. The emission wavelength of the SiNOH laser was 594 nm and a full width at half maximum (FWHM) linewidth of better than 1.5 nm limited by the spectrometer resolution was achieved. The output polarization was TM-like with a polarization ratio better than -20 dB. No spurious pump light was observed in the emission spectrum at the pump wavelength of 450 nm. Lasing was not achieved for a cavity without a slot-waveguide structure as the net gain is reduced compared to a slot-waveguide and the coupling to the lateral corrugation is decreased because of a stronger confinement of the waveguide mode.

**Figure 6:**
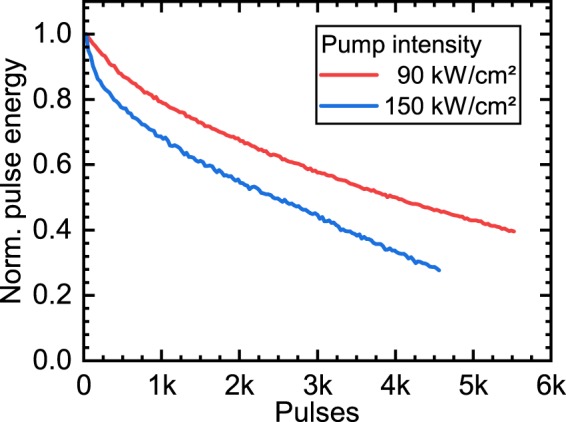
Decay of the SiNOH laser emission pulse energy over the number of optical excitation pulses. The pump intensity at 150 kW cm${}^{-2}$ is twice as high as the lasing threshold. The pump rate was 3 Hz at an optical pulse width of 35 ns.

The strong optical excitation of the gain material induces a degradation of the organic dye and limits the life time of the integrated light source. Figure 6 shows the decay curves of the SiNOH laser emission for a pump intensity of 90 kW cm${}^{-2}$ and 150 kW cm${}^{-2}$, respectively. If the device is operated two times above the lasing threshold, i.e. with a pump intensity of 150 kW cm${}^{-2}$, the emission pulse energy is reduced to the half of its initial value after $2400$ pulses. The device was operated at room temperature without an oxygen barrier. Oxygen barriers based on glass bonding have been shown to increase the life-span of OSSLs^47^ by a factor of up to $2500$. Together with an optimization of the lasing threshold, the operation lifetimes of SiNOH lasers are expected to be well suited for disposable sensing devices. Alternative dye molecules, e.g. Disperse Orange 11, with recoverable photodegradation properties^48^ and conjugated polymers^49^ could be evaluated for their photostability in the presented slot-waveguide SiNOH DFB laser geometry.

## Conclusion

The presented integrated silicon nitride organic hybrid laser light source operating in the visible wavelength region at 600 nm has the potential for a variety of applications in co-integrated sensing devices, where CMOS electronics and an integrated organic solid-state light source are combined. The direct in plane generation of laser light in a slot-waveguide mode facilitates highly sensitive PIC designs, e.g. slot-waveguide Mach-Zehnder interferometer. The polarization of the pump light influences the lasing threshold of the SiNOH laser. A threshold of 72 kW cm${}^{-2}$ has been achieved for a perpendicular pump polarization. Even though there is strong field enhancement in a slot-waveguide for the TE-like mode, the highest net gain is present for the TM-like mode and subsequently emission of the laser is consistently TM-like. However, for biosensing applications the TM like laser emission of the demonstrated SiNOH laser is an advantage over a TE-like polarization, since the elevated interaction with the evanescent field with the environment increases sensitivity for many applications.

The presented SiN-organic hybrid light source allows in principle for a local deposition of the gain medium via inkjet printing technology. This fabrication technique would reduce material usage, subsequently lowering costs and reducing environmental impact of waste and volatile organic compounds. With the added flexibility, different dye-doped polymer solutions could be incorporated into a single PIC to realize a co-integration of multiple independent light sources with different emission wavelengths.

## Methods

### Sample fabrication

The buried oxide (BOX) layer of 5 µm thickness and the silicon nitride layer of 160 nm were deposited on a silicon wafer by a plasma enhanced chemical vapour deposition (PECVD) process. The second order Bragg gratings and waveguide structures were defined by a 248-nm deep-ultra-violet (DUV) photolithography step and subsequent dry etching. A lift-off process with 4 µm sputtered silicon dioxide forming the top oxide (TOX) created openings to locally access the Bragg gratings with the gain material. Deep trenches etched into the substrate at the sample edge facilitated the end-face fiber coupling to the integrated strip-waveguides while minimizing back reflection. The fabrication of the samples was designed to be fully CMOS compatible for future applications with co-integrated electronics.

### Estimation of threshold gain

The coupled wave theory (CWT) including contributions of higher order partial waves^50^ was used to determine the coupling coefficients of the DFB grating. The resulting coefficients describe the coupling strength between the forward and backward propagating mode, and include radiative loss. The refractive indices for simulation were obtained from ellipsometry measurements, resulting in ${n}_{{\rm{BOX}}}=1.461$, ${n}_{{\rm{TOX}}}=1.485$, ${n}_{{\rm{SiN}}}=1.938$ at a wavelength of 600 nm, and from the datasheet of the polymer with ${n}_{{\rm{PMMA}}}=1.50$. Spectral gain and threshold gain values are given in units of power amplification rather than field amplification. The field amplification is half of the power amplification, e.g. $2{\alpha }_{{\rm{field}}}=\alpha$.

### Slot-waveguide propagation loss

The propagation loss for the slot-waveguide structure was determined at a wavelength of 633 nm with a helium-neon laser. The laser light was end-facet coupled to the PIC with a PM-fiber in TM-like polarization, divided on the chip with a Y-branch into a reference path and measurement path. The paths were routed to the output facet of the chip and coupled into a SM-fiber to measure the optical power with a power meter. The reference path employed a strip-waveguide, whereas the measurement path had a slot-waveguide section, including mode converter to change between strip- and slot-waveguide modes. Three different lengths (100 µm; 1000 µm; 1000 µm) of the slot-waveguide section were investigated. The propagation loss of the reference waveguide section was estimated from results obtained from large radii spirals of different lengths. For the mode converter from the slot-waveguide laser mode to the strip waveguide mode an insertion loss of 6.5 dB was determined.

### Organic dye PMN and dye-doped polymer

The synthetization of the organic dye molecule 2-(4-(bis(4-(*tert*-butyl)phenyl)amino)benzylidene)malononitrile (PMN) and the optimization of the dye concentration in the host matrix are described in a previous work^45^. Poly(methyl methacrylate) (PMMA) granulate (Polycasa, Acryl G77) was dried at 60 ${}^{\circ }{\rm{C}}$ for 1 h and then dissolved in anisole together with the organic dye PMN. The solution was filtered with a 200 nm pore size filter and spin-coated on the samples to form a homogeneous film of (500 ± 20) nm thickness. The solvent was removed with a soft backing step on a hot plate for 1 min at 110 ${}^{\circ }{\rm{C}}$. No deterioration of the dye from the backing step was observed. The doping level in the resulting polymer film was set to $5.0$ wt%, which results in highest material gain for the dye-doped polymer before concentration quenching sets in^45^. The absorbance and emission spectra were determined with dye-doped polymer coated microscope cover glasses in a UV-VIS spectrometer. The power attenuation coefficient $\mu$ for the dye-doped polymer is derived from the polymer film thickness and measured absorbance. The excitation wavelength of the spectrometer for the measurement of the emission spectrum was set to the wavelength of the pump source laser diode, i.e. 450 nm. The spectral modal gain of the slot-waveguide was determined with the variable stripe length (VSL) method^51^, where amplified spontaneous emission (ASE) light of different pump stripe lengths was coupled to a CCD-spectrometer via a single-mode fiber. The single-mode fiber ensured coupling of the waveguide mode only and avoided the spurious detection of fluorescence light propagating in the cladding. Data was fitted against the pump stripe length dependent ASE function $A/g\left(\exp (gL)-1\right)$, where $g$ is the modal gain, $L$ the pump stripe length, and $A$ a proportionality factor^51^.

### Optical excitation

**Figure 7:**
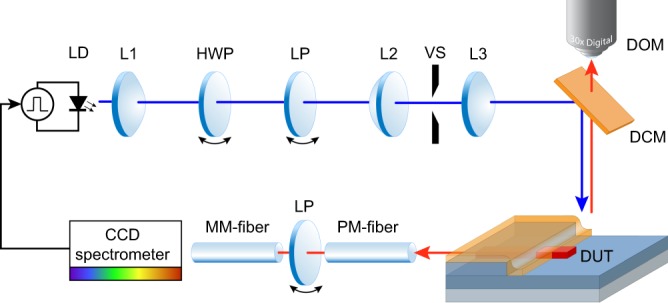
Measurement setup to pump the SiNOH DFB laser with a laser diode. Optical pulses are generated by a pulsed current source driving a 450 nm high power laser diode (LD). The combination of a half wave plate (HWP) and a linear polarizer (LP) acts as a variable attenuator. An intermediate image of the laser diode facet is created, where a variable slit (VS) is placed to truncate the pump spot length. This intermediate image is focused onto the sample (DUT) via a dichroic mirror (DCM). Visual inspection of the pump position is possible through a digital optical microscope (DOM).

Optical excitation of the organic gain medium was achieved by a high power multimode 450 nm blue laser diode driven with a pulsed current source. The resulting optical pulse width was 35 ns at a peak drive current of 38 A. The temporal shape of the optical pulse was verified with a 1 GHz silicon photodiode and a digital sampling oscilloscope. A combination of a half-wave plate and a linear polarizer acted as a variable attenuator for threshold measurements. The power of the pump beam was measured with an optically attenuated (OD3) free space silicon photodiode. The dimensions of the pump spot were measured with a digital microscope resulting in 400 µm $\times$ 25 µm. Figure 7 depicts the measurement setup. The measurement setup generates the elongated pump stripe from the emission characteristics of the laser diode without the need for a cylindrical lens. A variable slit is placed at the position of the intermediate image and used to truncate the pump spot length for VSL method modal gain measurements. The application of an intermediate image reduces spurious gain from Fresnel diffraction^52.^

### Measurement of laser emission

The laser light emission confined in the single-mode silicon nitride strip-waveguide was routed to the edge of the chip employing low-loss waveguide bends^53^ and coupled to a fiber via end-face coupling. For spectral measurements and determination of the threshold a multi-mode fiber (NA 0.20, 50 µm) was employed, whereas an polarization maintaining fiber (NA 0.12, 3.3 µm) was used to measure the polarization state of the integrated laser light source. The slow axis of the PM-fiber was aligned parallel to the sample edge. To determine the SiNOH laser pulse power, the CCD-spectrometer was calibrated against the known power of of a fiber-coupled 633 nm helium-neon laser.

### Data analysis and statistics

Spectral gain was obtained by a non-linear fit on filtered data (moving average with 7 nm window size). The $95 \%$ confidence band from the fit is shown together with the resulting gain. Lasing thresholds were determined for five measurements and error bars show the standard deviation.

## Data Availability

The authors declare that relevant data supporting the findings of this study are available on request.
